# The astrocytic sigma-1 receptor constitutes in the fast antidepressant action of hypidone hydrochloride (YL-0919) in rodents

**DOI:** 10.3389/fphar.2025.1564851

**Published:** 2025-03-26

**Authors:** Jin-Feng Li, Hai-Xia Chang, Jia-Ning Zhao, Jin-Hao Bao, Wei Dai, Yun-Feng Li

**Affiliations:** ^1^ Beijing Institute of Basic Medical Sciences, Beijing, China; ^2^ School of Life Sciences, Tsinghua University, Beijing, China; ^3^ Beijing Institute of Pharmacology and Toxicology, Beijing, China; ^4^ Shenyang Pharmaceutical University, Shenyang, China; ^5^ School of Basic Medical Sciences, Capital Medical University, Beijing, China

**Keywords:** astrocytes, sigma-1 receptor, antidepression, YL-0919, brain derived neurotrophic factor (BDNF)

## Abstract

**Introduction: **There is increasing evidence that astrocytes are involved in the therapeutic action of antidepressants. The fast antidepressant YL-0919 may interact with activation of astrocytic sigma-1 receptors (sigma-1R).

**Methods:** In this study, function of astrocytic sigma-1R in ventral hippocampus (vHIP) mediating the rapid antidepressant effect of YL-0919 were investigated. Adeno-associated virus (AAV) expressing shRNA was constructed to knock down astrocytic sigma-1R in vHIP, and the role of astrocytic sigma-1R on the rapid antidepressant action of YL-0919 were tested in chronic restraint stress (CRS) model of mice. Small interfering RNA (siRNA) was used to knock down sigma-1R in primary astrocytes, and we explored the mitochondrial function and BDNF expression of primary astrocytes after YL-0919 and siRNA treatments.

**Result: **The results indicated knocking down astrocytic sigma-1R in vHIP induced anxiety-like and depressive-like behavior in mice, and blocked the rapid anti-depressant and anxiolytic effects of YL-0919. Knocking down sigma-1R in primary astrocytes inhibited the YL-0919 induced enhancement of mitochondrial function and increased level of BDNF expression. In addition, increased BDNF in vHIP might play a role in fast antidepressant impact of YL-0919. Taken together, the data provide further evidence for a role of astrocyte receptors in the mechanisms of action of antidepressants.

**Conclusion:** Taken together, these results reveal increased BDNF in vHIP by affecting glial cells might be one of the significant mechanisms of fast antidepressant effect of YL-0919. The data provide further evidence for a role of astrocyte receptors in the mechanisms of action of antidepressants.

## 1 Introduction

Depression is a ubiquitous mental disorder with principle clinical symptoms of anhedonia and depressed mood. According to data from the World Health Organization, there are approximately 322 million people suffering from depression worldwide (Malhi and Mann, 2018). With the spread of the COVID-19 pandemic, the number of depression patients has surged to 53 million, which poses a severe challenge to the diagnosis and treatment of depression (Collaborators, 2021). However, the pathogenesis of depression is still not fully understood, and drug therapy is the major way for antidepressant treatment at present. The first-line antidepressant drugs, such as selective serotonin reuptake inhibitors (SSRIs) generally have defects of slow onset (3–6 weeks), low efficacy (50%–70%), and sexual dysfunction (Haase and Brown, 2015; Moncrieff et al., 2023). Therefore, the development of new antidepressants with rapid onset and low side effects remains a global urgent demand.

Astrocytes are the most numerous cell type in the central nervous system and primarily mediates energy supply to neurons, neurotransmitters recycling, ion homeostasis, immune responses and synaptic transmission (Stobart et al., 2018; Diaz-Castro et al., 2021; Kruyer et al., 2023). Pathologic changes of astrocytes are associated with multiple neuropsychiatric diseases, and receptors expressed on astrocytes may also be involved in the development and treatment of depression (Lee et al., 2022). Sigma-1R has been perceived as a potential target for therapy of neuropsychiatric diseases (Nguyen et al., 2015). Sigma-1R, a kind of low molecular weight protein, is mainly located in the endoplasmic reticulum and mitochondrial associated membrane regions. As a molecular chaperone protein, sigma-1R regulates many significant cellular biological processes including neurotransmitter release, inflammation, cell survival, autophagy and synaptogenesis (Nguyen et al., 2015). Besides neurons, sigma-1R is also highly expressed in astrocytes, and is closely related to modulate astrocytic calcium activity, mitochondrial function, brain derived neurotrophic factor (BDNF) release, inflammatory response and anti-oxidative stress (Ruscher et al., 2011; Mysona et al., 2018; Choi et al., 2022). Previous studies indicated that high expression of sigma-1R was detected in astrocytes of hippocampus dentate gyrus and CA3 region, and activation of astrocytic sigma-1R could promote cellular ATP production and neurogenesis in the hippocampus (Eraso-Pichot et al., 2017). In addition, sigma-1R activation protected against hydrogen peroxide (H2O2) induced oxidative stress damage and alleviated lipopolysaccharide (LPS) induced inflammatory responses in astrocytes (Weng et al., 2017). Sigma-1R also directly mediated astrocyte activation by affecting the extracellular signal-regulated protein kinases 1/2 (ERK1/2) and the glycogen synthase kinase 3β (GSK3β) signaling pathway in primary cultured astrocytes (Moon et al., 2014). *In vivo* specific activation of sigma-1R could improve the depressive behavior of chronic unpredictable mild stress (CUMS) mice, suggesting that astrocytic sigma-1R might become a potential target for depression treatment (Wang et al., 2020).

Hypidone hydrochloride (YL-0919), as a new antidepressant drug, is with developed by our institute with novel chemical structure, and is currently being tested in Phase II clinical trials (Li, 2020). It was found that YL-0919 exhibited rapid and significant antidepressant effects in different animal depression models and particularly performed the cognitive-enhancing impact as well. YL-0919 was proved to have a relatively high affinity for sigma-1R (Ren et al., 2023). The rapid antidepressant activity of YL-0919 (administered for 3 days) in rodents could be attenuated by pre-treatment with the selective sigma-1R antagonist BD-1047 (Ren et al., 2023). However, the mechanism underlying the fast antidepressant impact of YL-0919 is not well clarified, especially the role of astrocytes on action of YL-0919 remains unknown. The involvement of astrocytic sigma-1R in depression and the function of astrocytic sigma-1R mediating the rapid antidepressant effect of YL-0919 were investigated in this study.

AAV expressing shRNA was constructed to specifically knock down astrocytic sigma-1R in bilateral vHIP of mice, and we tested the role of astrocytic sigma-1R on the rapid antidepressant action of YL-0919. Since sigma-1R regulated mitochondrial function and BDNF synthesis, which were relevant to treatment for depression. siRNA was constructed to knock down sigma-1R in primary astrocytes, and mitochondrial function and BDNF expression of primary astrocytes after YL-0919 and siRNA treatments were investigated to explore the possible astrocytic mechanism of YL-0919. Finally, BDNF expression in vHIP was detected to verify its involvement in the rapid antidepressant effect of YL-0919 in the CRS model of mice.

## 2 Materials and methods

### 2.1 Animals

Male C57BL/6 mice (18–20 g) and postnatal mice within 1 day were purchased from Beijing SPF Biotechnology (China). The animals were housed 5 per cage under a 12-h light/dark cycle (lights on between 07:00 and 19:00) at appropriate temperature (22°C ± 2°C). Animals had free access to food and water. The mice were acclimated for 1 week before AAV injections or behavioral tests. All experimental procedures were approved by the institutional committee and performed in compliance with the National Institutes of Health Guide for the Care and Use of Laboratory Animals (ethical approval number: IACUC-DWZX-2022-605).

### 2.2 Drugs and reagents

YL-0919 (white powder, purity≥99.8%) was provided from Beijing Institute of Pharmacology and Toxicology. Fluoxetine was purchased from Sigma-Aldrich (#56296-78-7). Both YL-0919 (2.5 mg/kg) and fluoxetine (10 mg/kg) were dissolved in sterile physiological saline, and the dose of YL-0919 and fluoxetine was selected as previous studies (Manosso et al., 2013; Ren et al., 2023). The mice were administered intragastrically (i.g.) with the drugs at a volume of 10 mL/kg.

### 2.3 Stereotaxic viral injection

The AAV expressing shRNA (pAAV-GfaABC1D-EGFP-3 × FLAG-miR30shRNA (Sigmar1)-WPRE) for specific knockdown of astrocytic sigma-1R was constructed and generated by BrainVTA Co., Ltd., (Wuhan, China). The target sequences of shRNAs were presented as following: GAC​TAT​TAT​CGC​AGT​GCT​GAT (1#); CCT​GTA​GTA​ATC​TCT​GGT​GAA (2#); GCG​TAT​ACC​ATG​CAG​ATA​TTA (3#). Mice were anesthetized with 1% sodium pentobarbital. The scalp was sterilized, and the skin was open to remove excess periosteum tissues. The mice were micro-injected with a total of 0.5 μL AAV expressing shRNAs (>1 × 10^12^ gene copies/mL) or the scramble virus (control group) bilaterally in the vHIP region (AP: –2.8, ML: ±1.5, DV: −4.0 mm, The Mouse Brain in Stereotaxic Coordinates, Second Edition). The delivery rate of injection was 0.05 μL/min by an ultra-micro injection pump (RWD, China). After AAV administration, the needle remained in place for 10 min and was carefully retracted to avoid backflow. Immunofluorescence staining, Western blot and behavioral tests were performed successively 4 weeks after the AAV injection.

### 2.4 Immunofluorescence for brain sections

The mice were anesthetized and transcardially perfused with 4% paraformaldehyde (PFA). The brains were removed after perfusion and fixed with 4% PFA for 2 days. Then the brains were cryoprotected in 30% sucrose for 3 days. The brain sections (30 μM) were made by using a cryostat (Leica, # CM3050S) at −20°C, and washed three times with PBS. The sections were blocked in PBS containing 0.3% Triton-X-100, 5% goat serum and 2% BSA for 1.5 h at room temperature, followed by incubation at 4°C overnight with following primary antibodies respectively: anti-GFAP ccc (Millipore, MAB360, 1:500); anti-Iba1 (NOVUS, NB100-1028, 1:500) and anti-Neun (Cell signaling technology, 94403S, 1:500). On the second day, the sections were incubated with donkey anti-mouse and donkey anti-goat secondary antibody (Jackson ImmunoResearch) individually for 1 h at room temperature in the dark. Images were acquired by using a Nikon confocal microscope.

### 2.5 CRS paradigm

The CRS mice were individually placed into a modified transparent and well-ventilated centrifuge tube (50 mL, NEST Biotechnology) for 6 h per day (from 10 a.m. to 16 p.m.) for 14 consecutive days. The mice were only permitted subtle movements, and were not able to move forward or backward in the tubes. After CRS paradigm, mice were administered daily by YL-0919 (2.5 mg/kg, i.g.), fluoxetine (10 mg/kg, i.g.) and vehicle separately for 5 consecutive days. The procedures of drug treatments and behavioral tests were demonstrated in Figure 4A.

### 2.6 Open field test (OFT)

The mice were acclimated in the experimental room for 1 h before tests. The apparatus was consisted of four opaque white arenas (50 cm × 50 cm × 20 cm). The mice were individually placed in the center of the open field arena, and allowed to explore freely for 15 min. The total distance traveled and the duration spent in the center zone (25 cm × 25 cm) of the last 10 min were recorded with video and automatically quantified by software (ANY-maze, Stoelting, United States) for statistical analysis.

### 2.7 Elevated plus maze test (EPM)

Before testing, animals were acclimated in the experimental room for 1 h. The elevated plus maze was composed of four branching arms (two open arms and two enclosed arms, 30 cm × 30 cm), and collected by a central area (5 cm × 5 cm). The height of the elevated plus maze was 50 cm. Mice were placed individually into the junction of the branching arms (facing to one of the open arms) and allowed to explore for 6 min. The activity of the mice in the elevated plus maze was recorded by SMART video acquisition system, and percentage of time spent in open arms was quantified and analyzed.

### 2.8 Novel object recognition test (NORT)

The mice were acclimated in the experimental room for 1 h, and were tested in two non-transparent open fields (50 cm × 50 cm × 20 cm). In the adaptation session, Mice were individually placed within the arena and were allowed to explore freely for 10 min. On the second day of the test, the mice were allowed to explore two identical objects in the same transparent open fields for 10 min. Then, an hour later, one of the familiar objects was replaced by a novel object in retention trial of the test, and the mice were reintroduced to the same arena for 5 min. The cumulative time of exploring familiar and novel objects were recorded separately. A NOR index was calculated as exploration time for novel object/(exploration time for novel object + exploration time for familiar object).

### 2.9 Forced swim test (FST)

After being acclimated in the experimental room for 1 h, the mice were individually placed to swim in glass cylinders (height: 25 cm, diameter: 18.5 cm) containing water with a depth of 15 cm at controlled temperature (24°C ± 1°C). The animals remained in the water for 6 min, and the immobility time of mice for the last 4 min was measured. Immobility duration was defined as the time when the animals performed minimal movements required to keep their heads above the water.

### 2.10 Sucrose preference test (SPT)

Before test, all mice were housed singly and acclimated with two bottles of 1% (w/v) sucrose solution for 24 h. Then the mice were individually exposed to one bottle of 1% (w/v) sucrose solution and one bottle of drinking water for 12 h in the dark. After 6 h, two bottles’ positions were exchanged to avoid place preference. The bottles were weighed before and after each test, and sucrose preference was calculated as followed formula. The sucrose preference index (%) = Consumption of sucrose solution/Consumption of sucrose solution plus water×100%.

### 2.11 Tail suspension test (TST)

The mice were acclimated in the experimental room for 1 h before testing. Mice were individually suspended by the secured tails from a ledge with adhesive tapes (approximately 1 cm from the tip of the tail) for 6 min. Mice were monitored and recorded using a video camera system, and the immobility time of the last 4 min was measured.

### 2.12 Primary astrocyte culture

Primary astrocyte cultures were prepared from newborn C57BL/6 mice within 24 h. The brains were dissected without olfactory bulb and cerebellum, and surface meninges were removed. The tissues were collected and digested in 0.25% Trypsin containing 0.1% DNase I at 37°C for 15 min with occasional swirling. The digestion was finished with isopycnic DMEM: F12 medium (containing 10% fetal bovine serum and 1% Pen-Strep antibodies), and the supernatant containing dissociated cells was passed through a 70 μm nylon mesh cell strainer. Then the filtrate was centrifuged at 1,500 rpm for 10min to pellet the cells. The supernatant was removed and cells were resuspended in medium. The cells were seeded in plates coated with poly-D-lysine and cultured in an incubator (5% CO_2_) at 37°C. After 7 days’ culture, primary microglia were removed by a slight bump, and the astrocytes were cultured for 14 days prior to use.

### 2.13 siRNA transfection

The siRNAs for knockdown of sigma-1R was constructed by Shanghai GenePharma Co., Ltd. Transfection of siRNA in primary astrocytes was performed by using lipofectamine™ RNAiMAX (Invitrogen) as the manufacturer’s instructions. Primary astrocytes were cultured in 24-well plates at approximate 80% confluency. The culture media was replaced by antibiotic-free glia media before transfection. Then transfection reagent as mixtures of Opti-MEM, lipofectamine, and siRNAs were prepared and incubated at room temperature for 15–20 min and added drop wise to the astrocytes for transfection of each well. 48 h post transfection, the transfection reagent was replaced by complete medium, and efficiency of siRNA for sigma-1R knockdown was tested by Western blot.

### 2.14 ATP measurement

ATP measurements were determined by using a bioluminescent ATP assay kit (Promega, #G7573) according to the manufacturer’s instructions. Cells were seeded and cultured onto 96-well plates overnight before tests. Then the cells were treated with YL-0919 (final concentration 2 μM) or vehicle for 1 h, and 50 μL of supernatant was transferred to another 96-well plate and added with detection reagent. After incubation for 10 min at room temperature in the dark, the extracellular ATP level was determined by using a microplate reader. For intracellular ATP level measurement, the remaining supernatant was discarded, and 100 μL of lysis buffer containing luciferase reagents was incubated for 10 min in the dark at room temperature. The fluorescence intensity of intracellular ATP was also tested using the microplate reader. For quantification, total protein levels of cells were used for normalization.

### 2.15 Measurement of mitochondrial membrane potential

Mitochondrial membrane potential (ѱ_m_) was assayed by using tetramethylrhodamine methyl ester assay kits (TMRM, Invitrogen, I34361). Primary astrocytes were seeded onto 6-well plates and loaded with 50 nM TMRM for 30 min at 37°C in the dark. The TMRM reagent was removed, and astrocytes were washed with PBS for 3 times. Then the cell suspension was prepared by trypsinization. TMRM accumulates in cell mitochondria, and signal intensity of TMRM represents the function of membrane potential. The mean fluorescence intensity (MFI) was measured and analyzed by flow cytometry.

### 2.16 Western blot

Hippocampal tissue or primary astrocytes were sonicated and homogenized in radio immunoprecipitation (RIPA) assay buffer containing protease inhibitors and phosphatase inhibitors. The lysed tissues were centrifuged, and the supernatants were collected. A total of 30 μg of each protein sample was loaded onto SDS-polyacrylamide gel electrophoresis gels, and transferred to 0.45 μm polyvinylidene difluoride membranes. The membranes were blocked with 5% skim milk for 1 h at room temperature, and incubated with the following primary antibodies against sigma-1R (1:1,000, Cell signaling technology, 61994S), pro-BDNF (1:1,000, Santa Cruz, sc-65514), BDNF (1:1,000, Abcam, ab108319) and β-actin (1:1,000, Proteintech, 60008-1-1) individually at 4°C overnight. Then secondary antibodies (1:5,000, Jackson ImmunoResearch) were incubated for 2 h at room temperature. Images was visualized by using an Odyssey two-color infrared imaging system.

### 2.17 Statistical analysis

All data are presented as Means ± SEMs. The significance of differences was analyzed using Student’s t-test, one-way ANOVA followed by Dunnett’s test or two-way ANOVA followed by Tukey’s multiple comparisons test. The *P* < 0.05 was considered statistically significant.

## 3 Results

### 3.1 Sigma-1R knockdown in vHIP induced depressive and anxiety-like behaviors in mice

**FIGURE 1:**
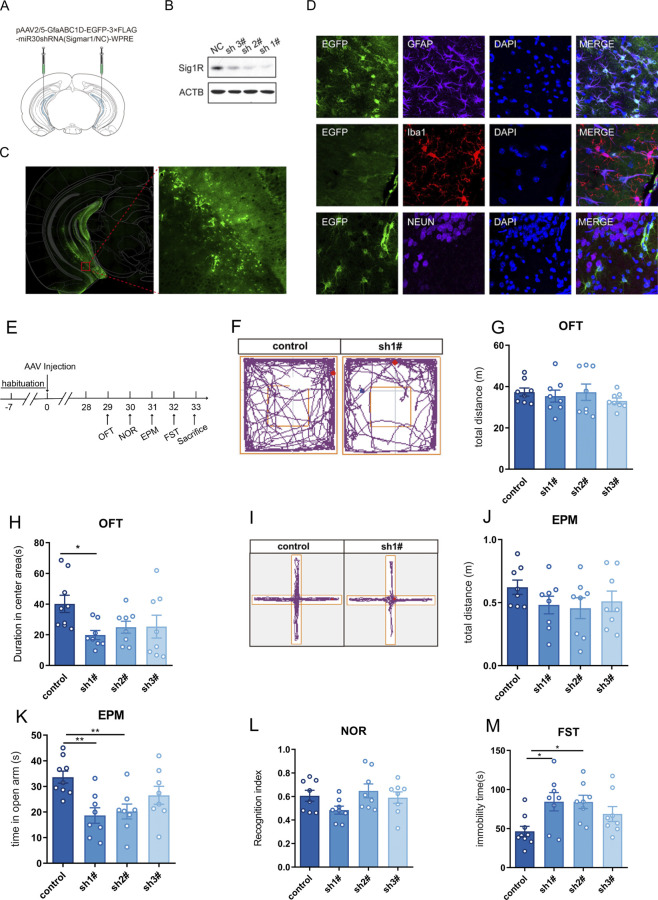
Knockdown of Sigma-1R in vHIP induced depressive and anxiety-like behaviors in mice. **(A)** Experimental schematic of AAV injection; **(B)** Western blotting assays for detecting the expression level of Sigma-1R; **(C)** Immunofluorescence staining of EGFP for detecting the location of AAV infection; **(D)** Immunofluorescence staining of GFAP, Iba1 and Neun were performed to detect that AAV carrying shRNA was specific for astrocytes; **(E)** Experimental schematic to assess the behavior of mice with AAV injection; **(F)** Representative trace recordings of OFT; **(G)** total distance moved in the OFT; **(H)** duration spent in the center area of OFT; **(I)** Representative trace recordings of EPM; **(J)** total distance moved in EPM, **(K)** time spent in open arms of EPM; **(L)** Recognition index of mice; **(M)** immobility time of FST. Data are expressed as mean ± SEM, n = 8–9, ^*^Significant difference by one-way ANOVA, followed by Dunnett’s test, ^*^ *P* < 0.05; ^**^ *P* < 0.01.

To investigate the impact of astrocytic sigma-1R knockdown in vHIP on the behavior of mice, the AAV vector carrying shRNA targeting the sigma-1R gene under the direction of the astrocyte-specific promoter GfaABC1D were micro-injected bilaterally in the vHIP regions (Figure 1A). 4 weeks after the AAV vector injection, Western blot test suggested shRNAs successfully suppressed sigma-1R protein expression in vHIP, and AAV carrying shRNA 1# revealed the most significant knockdown efficacy (Figure 1B). AAV carrying shRNA 1# was then performed in the following tests. Immunofluorescence studies showed that EGFP fluorescence was primarily observed in vHIP (Figure 1C) and the expression of AAV was specific for astrocytes (co-staining of GFAP^+^ cells and EGFP^+^cells) rather than neuron (Neun^+^ cells) and microglia (Iba1^+^ cells) in vHIP (Figure 1D).

We next examined the effects of astrocytic sigma-1R knockdown in vHIP on the behavior of mice (Figure 1E). The spontaneous locomotor activity was investigated in the OFT, and no significant difference was found between the AAV carrying shRNA group and control group (Figures 1F, G, F (3, 28) = 0.5371, P > 0.05). AAV carrying shRNA1# injected mice had significantly lower duration spent in the center area than controls (Figure 1H, F (3, 29) = 2.859, P < 0.05), and AAV carrying shRNA1#/2# treated mice showed significantly decreased time spent in open arms compared to control groups (Figure 1K, F (3, 29) = 5.569, P < 0.01). Behavioral assays suggested the anxiety-like phenotypes in mice with astrocytic sigma-1R knockdown. No significant difference of total distance in EPM was found between the AAV carrying shRNA groups and control group (Figures 1I, J, F (3, 28) = 1.003, *P* > 0.05). In addition, the injection of AAV carrying shRNAs had no impact on the NOR index of mice compared to control groups (Figure 1L, F (3, 28) = 2.132, *P* > 0.05). AAV carrying shRNA1#/2# injected mice had significantly higher immobility duration than controls (Figure 1M, F (3, 29) = 4.006, *P* < 0.05), indicating that the depression-like phenotypes in mice with astrocytic sigma-1 gene knockdown. Then AAV carrying shRNA1# was used in the following tests. Taken together, these results suggested astrocytic sigma-1R knockdown in vHIP of mice leading to depressive and anxiety-like behaviors.

### 3.2 Knockdown of astrocytic sigma-1R in the vHIP region inhibited the fast anti-anxiety and antidepressant effect of YL-0919 in mice

**FIGURE 2:**
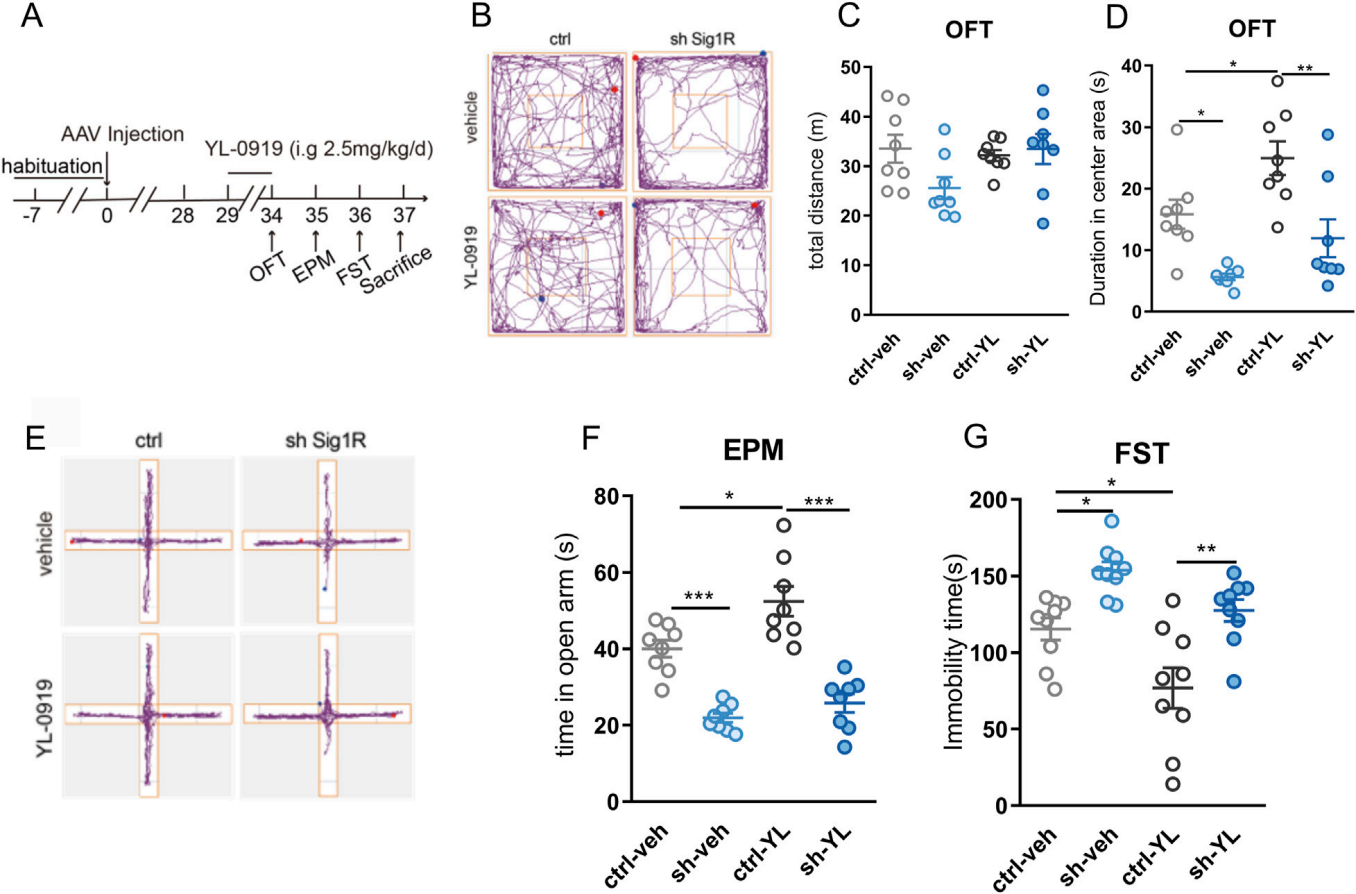
Specific knockdown of astrocytic sigma-1R in the vHIP blocked the fast anti-anxiety and antidepressant effect of YL-0919 in mice **(A)** Schematic diagram of drug administration and behavior tests; **(B)** Representative trace recordings of OFT; **(C)** total distance moved in the OFT; **(D)** duration spent in the center area of OFT; **(E)** Representative trace recordings of EPM; **(F)** time spent in open arms of EPM; **(G)** immobility time of FST. Data are mean ± SEM, n = 8, ^*^Significant difference by two-way ANOVA, followed by Tukey’s multiple comparisons test, ^*^ *P* < 0.05, ^**^ *P* < 0.01, ^***^ *P* < 0.001.

To assess the contribution of astrocytic sigma-1R on the fast anti-anxiety and antidepressant effect of YL-0919, behavioral tests were performed after 5 consecutive days of YL-0919 administration (2.5 mg/kg, i. g., Figure 2A). No significant difference was found in the spontaneous locomotor activity of OFT between the AAV carrying shRNA injection and YL-0919 treatment (Figures 2B, C, F (7, 21) = 1.947, *P* > 0.05). YL-0919 injected mice had significantly increased duration spent in the center area of OFT (Figures 2B, D; F (7, 21) = 1.384, control-veh vs. control-YL-0919, *P* < 0.05) and increased time spent in open arms of EPM (Figures 2E, F, F (7, 21) = 0.7176, control-veh vs. control-YL-0919, *P* < 0.05) compared to control groups, suggesting the fast anti-anxiety effect of YL-0919. Compared to shRNA-veh group, duration spent in the center area of OFT (Figures 2B, D; F (7, 21) = 1.384, shRNA-veh vs. shRNA-YL-0919, *P* > 0.05) and time spent in open arms of EPM (Figures 2E, F, F (7, 21) = 0.7176, shRNA-veh vs. shRNA-YL-0919, *P* > 0.05) had no change in shRNA-YL-0919 group, indicating that the fast anti-anxiety effect of YL-0919 was blocked by the pretreatment of knockdown of astrocytic sigma-1R. In addition, YL-0919 injected mice had significantly lower immobility duration of FST compared to control groups (Figure 2G, F (8, 24) = 0.8404, control-veh vs. control-YL-0919, *P* < 0.05), suggesting the fast anti-depression effect of YL-0919. Compared to shRNA-veh group, the immobility duration of FST remained unchanged in shRNA-YL-0919 group (Figure 2G, F (8, 24) = 0.8404, shRNA-veh vs. shRNA-YL-0919, *P* > 0.05), indicating that the fast anti-depression effect of YL-0919 was also inhibited by the pretreatment of knockdown of astrocytic sigma-1R. These results verified astrocytic sigma-1R in vHIP underlying the fast anti-depression and anti-anxiety impact of YL-0919.

### 3.3 The regulatory effects of YL-0919 on mitochondrial function and BDNF expression in primary astrocytes was blocked by knockdown of astrocytic sigma-1R

**FIGURE 3:**
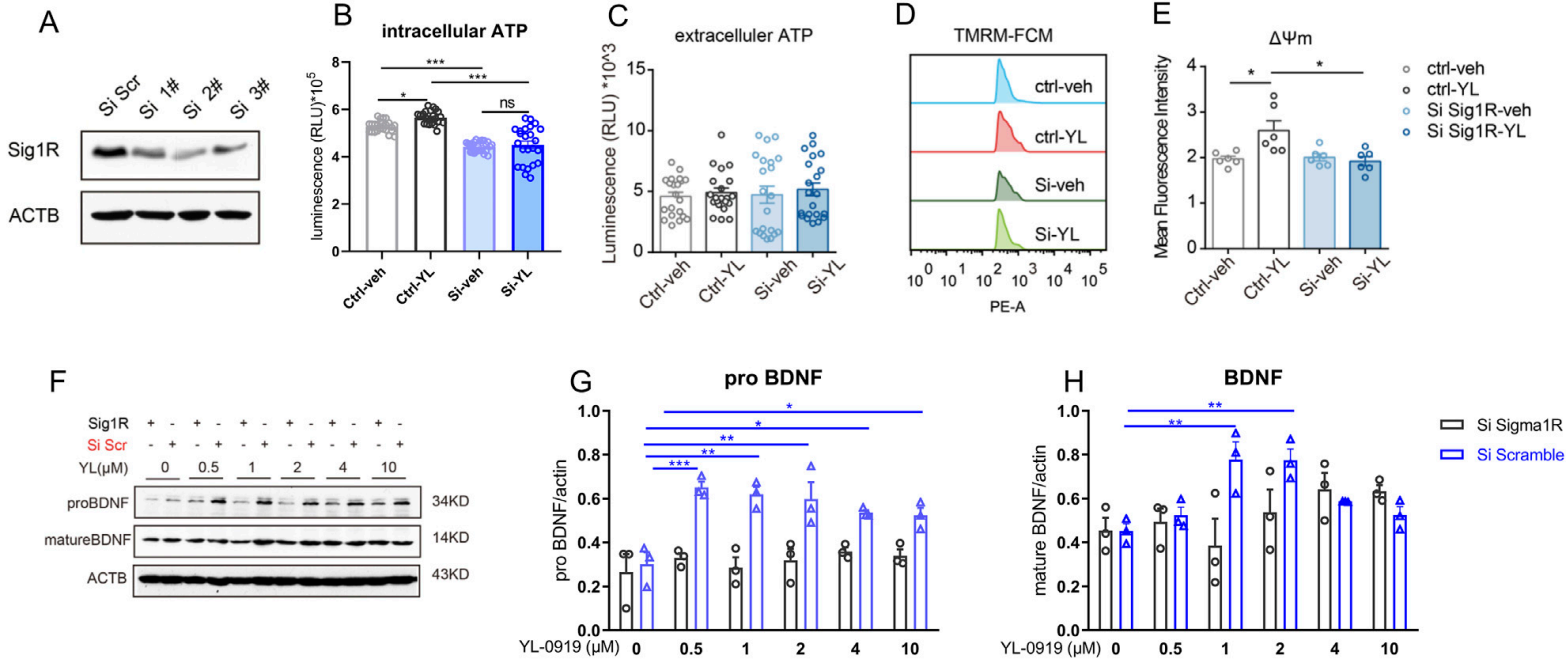
Knockdown of astrocytic sigma-1R blocked the regulatory effects of YL-0919 on mitochondrial function and BDNF expression in primary astrocytes **(A)** Western blotting assays for detecting the expression level of Sigma-1R in primary astrocytes; **(B, C)** Measurement of intracellular ATP and extracellular ATP, n = 21; **(D)** The mitochondrial membrane potential of primary astrocytes was measured by TMRM fluorescence; **(E)** mean fluorescence intensity of mitochondrial membrane potential in primary astrocytes, n = 6; **(F–H)** Western blot analysis of pro-BDNF and mature BDNF in primary astrocytes, n = 3; ^*^Significant difference by two-way ANOVA, followed by Tukey’s multiple comparisons test **(A–E)** and one-way ANOVA, followed by Dunnett’s test **(F–H)**, ^*^ *P* < 0.05, ^**^ *P* < 0.01, ^***^ *P* < 0.001.

The knockdown of sigma-1R in primary astrocytes were performed by siRNAs. siRNA 2# showed significant knock-down efficacy compared to control group (Figure 3A), and was then performed in the following tests. The astrocytic mitochondrial function was measured by ATP production and mitochondrial membrane potential. We found that knockdown of sigma-1R by siRNA significantly inhibited the intracellular ATP production in primary astrocytes (Figure 3B, F (3, 69) = 37.65, control-veh vs. siRNA-veh, *P* < 0.001). The intracellular ATP level of astrocytes was increased by 1 h pre-incubation of YL-0919 (2 μM) compared to control group (Figure 3B, F (3, 69) = 37.65, control-veh vs. control-YL-0919, *P* < 0.05), and the enhancement ATP level by YL-0919 was blocked by knockdown of sigma-1R by siRNA (Figure 3B, F (3, 69) = 37.65, siRNA-veh vs. siRNA-YL-0919, *P* > 0.05). siRNA or YL-0919 treatment exerted no impact on the released ATP by astrocytes (Figure 3C, F (3, 60) = 0.3684, *P* > 0.05). We next assessed the effect of sigma-1R knockdown and YL-0919 treatment on the mitochondrial membrane potential of primary astrocytes by TMRM fluorescence. Knockdown of sigma-1R by siRNA did not affect the mitochondrial membrane potential (Figures 3D, E, F (3, 15) = 5.211, control-veh vs. siRNA-veh, *P* > 0.05). 1 h pre-incubation of YL-0919 (2 μM) significantly upregulated mitochondrial membrane potential by increasing mean fluorescence intensity compared to control group (Figures 3D, E, F (3, 15) = 5.211, control-veh vs. control-YL-0919, *P* < 0.05), and the modulation of astrocytic mitochondrial membrane potential by YL-0919 was inhibited with knockdown of sigma-1R by siRNA (Figures 3D, E, F (3, 15) = 5.211, siRNA-veh vs. siRNA-YL-0919, *P* > 0.05). The results suggested knockdown of sigma-1R impaired the intracellular ATP production in primary astrocytes, and YL-0919 upregulated astrocytic mitochondrial function by sigma-1R activation.

The effect of sigma-1R knockdown and YL-0919 treatment on the astrocytic levels of pro-BDNF and mature BDNF were measured by Western blot. The primary astrocytes were treated with several doses of YL-0919 (0, 0.5, 1, 2, 4 and 10 μM) for 1 h, and astrocytes were collected 24 h after YL-0919 treatments for western blot (Figure 3F). Compared to control group, YL-0919 (0.5–10 μM) significantly increased the expression of pro-BDNF (Figure 3G, F (5, 12) = 7.741, YL-0919 (0.5–10 μM) + scramble vs. YL-0919 (0 μM) + scramble, *P* < 0.05) and YL-0919 (1 and 2 μM) also promoted the level of mature BDNF (Figure 3H, F (5, 12) = 8.674, YL-0919 (0.5–10 μM) + scramble vs. YL-0919 (0 μM) + scramble, *P* < 0.01). After knockdown of sigma-1R by siRNA, YL-0919 did not affect the expression of pro-BDNF (Figure 3G, F (5, 12) = 0.5529, YL-0919 (0.5–10 μM) + siRNA vs. YL-0919 (0 μM) + siRNA, *P* > 0.05) and mature BDNF (Figure 3H, F (5, 12) = 1.552, YL-0919 (0.5–10 μM) + siRNA vs. YL-0919 (0 μM) + siRNA, *P* > 0.05). The results indicated that YL-0919 upregulated astrocytic BDNF expression by sigma-1R activation.

### 3.4 BDNF in vHIP was involved in fast antidepressant effect of YL-0919

**FIGURE 4:**
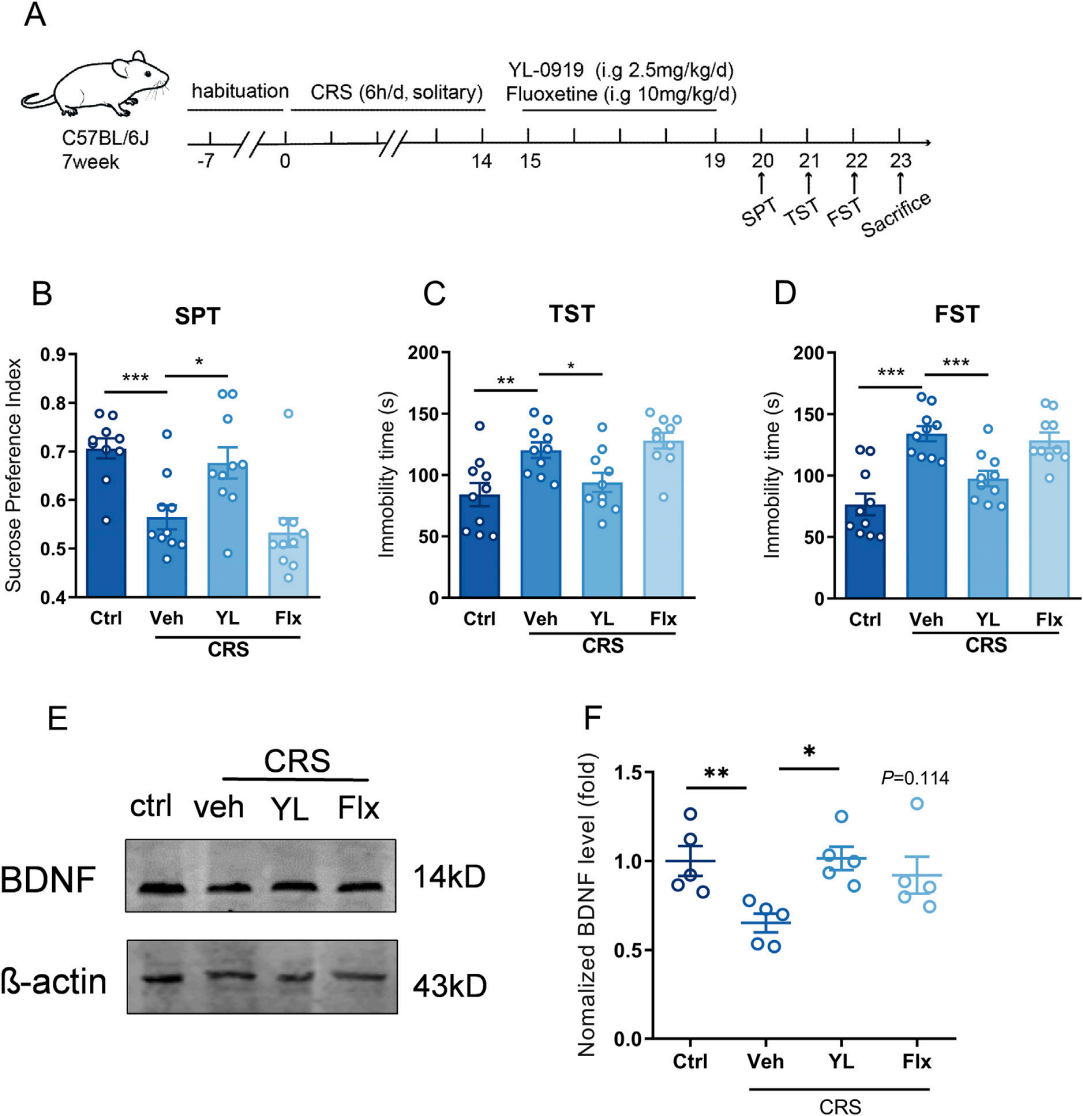
BDNF in vHIP contributed to the fast antidepressant effect of YL-0919 **(A)** Schematic diagram of fluoxetine/YL-0919 administration and behavior tests, n = 10; **(B)** The sucrose preference index of SPT; **(C, D)** immobility time of TST and FST; **(E, F)** Western blot analysis of BDNF expression in vHIP, n = 5, ^*^Significant difference by *t*-test and one-way ANOVA, followed by Dunnett’s test, ^*^ *P* < 0.05; ^**^ *P* < 0.01, ^***^ *P* < 0.001.

We found that astrocytic sigma-1R in vHIP constituted in the fast anti-depression impact of YL-0919, and YL-0919 increased astrocytic BDNF expression by sigma-1R activation. Then the involvement BDNF in vHIP in fast antidepressant effect of YL-0919 was verified. Compared to control group,14 days of CRS procedure induced the depressive-like behavior in mice for decreased sucrose preference index in SPT (Figure 4A, t (18) = 4.397, control + veh vs. CRS + veh, *P* < 0.001) and increased immobility durations in TST (Figure 4C, t (18) = 3.179, control + veh vs. CRS + veh, *P* < 0.01) and FST (Figure 4D, t (18) = 5.377, control + veh vs. CRS + veh, *P* < 0.001). Consecutive administration of YL-0919 for 5 days (2.5 mg/kg, i. g.) performed fast antidepressant effect for increased sucrose preference index in SPT (Figure 4B, F (2, 27) = 6.742, CRS + veh vs. CRS + YL-0919, *P* < 0.05) and decreased immobility duration in TST (Figure 4C, F (2, 27) = 6.761, CRS + veh vs. CRS + YL-0919, *P* < 0.05) and FST (Figure 4D, F (2, 27) = 10.01, CRS + veh vs. CRS + YL-0919, *P* < 0.001) compared to CRS-veh group. Consecutive administration of classical SSRI antidepressant fluoxetine for 5 days (10 mg/kg, i. g.) did not exert antidepressant impact compared to CRS-veh group (CRS + veh vs. CRS + Flx, Figure 4B, F (2, 27) = 6.742, *P* > 0.05; Figure 4C, F (2, 27) = 6.761, *P* > 0.05; Figure 4D, F (2, 27) = 10.01, *P* > 0.05), which was identified with relatively slow onset time (21 days) of fluoxetine from previous study. Expression of BDNF in vHIP was measured after behavior tests. The level of BDNF in vHIP was significantly reduced after CRS exposure compared to control group (Figures 4E, F, t(8) = 3.528, *P* < 0.01), and the decreased BDNF level was recovered with YL-0919 treatment (Figures 4E, F, F (2, 12) = 5.985, CRS + veh vs. CRS + YL-0919, *P* < 0.05) instead of fluoxetine administration (Figures 4E, F, F (2, 12) = 5.985, CRS + veh vs. CRS + Flx, *P* > 0.05). The data suggested that increased BDNF in vHIP was involved in fast antidepressant effect of YL-0919.

## 4 Discussion

YL-0919 has a significant and rapid antidepressant effect in different animal models, but whether astrocyte sigma-1R in vHIP contributes to action of YL-0919 is not known. This study found that knocking down astrocytic Sigma-1R in vHIP induced anxiety-like and depressive-like behavior in mice, and blocked the rapid anti-depressant and anxiolytic effects of YL-0919. Knocking down astrocytic sigma-1R in primary astrocytes inhibited the positive regulation of mitochondrial function and increased level of BDNF expression by YL-0919 treatment. Compared to fluoxetine, increased BDNF in vHIP by affecting glial cells might be one of the significant mechanisms of fast antidepressant effect of YL-0919.

Substantial evidence has identified that pathological changes of Hippocampal contributes to the episode and severity of depression (Frodl et al., 2008). The dorsal and ventral hippocampus of rodents mediates diverse functions. The dorsal hippocampus mainly adjusts cognitive ability, such as learning and memory, while vHIP is more closely related to stress and emotion regulation (Fanselow and Dong, 2010). Depressive disorder could lead to the reduced density, morphologic variations (enlarged cell bodies, and decreased complexity of branches, and functional disorders) and function abnormality in the prefrontal cortex and hippocampus of mice (Murphy-Royal et al., 2019; Codeluppi et al., 2021). Astrocytes might be involved in the rapid antidepressant effect of ketamine. Treatment with rapid antidepressant ketamine could increase the size of astrocytes as well as the number and length of astrocytic processes in the hippocampus of depression model of rats (Ardalan et al., 2017), indicating that hippocampal astrocytes might play a critical role in depression pathology and antidepressant treatment. Our study found that specific knockdown of astrocyte sigma-1R in the vHIP could induce significant depression and anxiety-like behaviors in mice, suggesting that astrocyte sigma-1R in vHIP participated in modulating animal emotion. Furthermore, knockdown of sigma-1R in astrocytes of vHIP also blocked the anti-depressive and anti-anxiety effect of YL-0919, suggesting that astrocytic sigma-1R in vHIP played significant role in the rapid antidepressant action of YL-0919.

Mitochondria are the main organelles that produce energy in eukaryotic cells. Increasing evidences suggested that mitochondrial dysfunction was related to the pathogenesis of depression (Simon et al., 2021). Abnormal mitochondrial function increased the production of inflammatory factors, and aggravated cerebral neuroinflammation and depressive-like behaviors in rodents (Allen et al., 2021). Astrocyte mitochondrial dysfunction occurs in a variety of neuropsychiatric diseases as well. The dysfunction of astrocytic mitochondrial oxidative phosphorylation is manifested as the characteristics of cognitive impairment and anxiety (Mi et al., 2023). Mitochondria are best known as the sites for production of respiratory ATP, and ATP can act as a glial transmitter to regulate neuronal homeostasis, while insufficient ATP can lead to impaired synaptic plasticity (Illes et al., 2020). Promoted ATP release from hippocampal astrocytes regulates synaptic activity of DG granule cells, and alleviates anxiety-like behavior in mice (Cho et al., 2022). Thus, mitochondrial function and ATP synthesis and release of astrocytes may play an important role in depression. Our previous study found that genes related to mitochondrial function in astrocytes of the mouse brain changed dramatically on gene-set enrichment analysis of RNA-seq after YL-0919 administration, and upregulated oxidative phosphorylation level of primary astrocytes with YL-0919 treatment was also verified by Seahorse test. The results further verified that YL-0919 treatment significantly increased astrocyte mitochondrial function by increasing intracellular ATP levels and elevating mitochondrial membrane potential. Besides, knocking down the sigma-1R in primary astrocytes reduced synthesis of intracellular ATP, and blocked the enhancing effects of YL-0919 on ATP level and mitochondrial membrane potential, suggesting that the sigma-1R receptor could be the target of YL-0919 in regulating astrocyte mitochondrial function. YL-0919 might improve astrocytic mitochondrial function by activating the sigma-1R receptor, thereby exerting an antidepressant effect.

Studies have shown that low level of BDNF is involved in the pathogenesis of depression (Buttenschon et al., 2015). Increasing BDNF synthesis and secretion may be an effective strategy for depression treatment by promoting the regeneration of neurons and glial cells (Cheon et al., 2018). Cerebral BDNF is mainly synthesized from neurons and glial cells (Illes et al., 2020). After pre-pro-BDNF in the endoplasmic reticulum is transferred to the Golgi apparatus, the signal sequence in the pre region is cleaved to form the precursor protein of BDNF (pro-BDNF). Then pro-BDNF is further cleaved to form mature BDNF (mature-BDNF). Pro-BDNF and mature-BDNF have different biological roles, and tissue-type plasminogen activator (tPA) is essential for the cleavage of BDNF precursor into mature BDNF (Hoirisch-Clapauch, 2022). The tPA and BDNF were significantly reduced in the serum of patients with depression. 8 weeks of treatment with escitalopram or duloxetine significantly elevated the levels of tPA and BDNF, suggesting that increased BDNF expression was closely related to the onset of antidepressants (Jiang et al., 2017). Activated astrocytes also synthesized and released BDNF, and overexpression of BDNF in hippocampal astrocytes rescued the depression and anxiety-like behaviors of mice. Facilitation of BDNF synthesis and release in hippocampal astrocytes might be a key mechanism for the rapid antidepressant effects (Quesseveur et al., 2013). This study found that YL-0919 (0.5–10 μM) significantly increased the synthesis of pro-BDNF in primary astrocytes, and relatively low dose of YL-0919 (0–2 μM) significantly increased the expression of mature-BDNF, which could be blocked by astrocytic sigma-1R knockdown, indicating that astrocytic sigma-1R mediated the enhancing effect of YL-0919 on BDNF expression. In the study, we also found that high-dose YL-0919 (4, 10 μM) did not promoted BDNF expression, and a bell-shaped dose-response curve might exist between biological effect of increasing BDNF and doses of YL-0919. Previous studies showed that ligand of sigma-1R increased cytoplasmic free calcium concentration in a bell-shaped manner, and calcium activity regulated most astrocytic functions (Hayashi et al., 2000; Guerra-Gomes et al., 2017). This might explain the nonlinear regulatory effect of YL-0919 on astrocytic BDNF expression.

After investigating that astrocytic sigma-1R in vHIP mediated the rapid antidepressant action of YL-0919, and YL-0919 upregulated BDNF by activating sigma-1R in glia cells, we further clarified the relationship between BDNF in vHIP and the rapid onset of YL-0919. The results manifested that YL-0919 administration for 5 consecutive days significantly improved the depressive-like behavior of CRS mice, and significantly increased the BDNF expression level in the vHIP. Whereas, the classic antidepressant fluoxetine did not exert an antidepressant effect after 5 days of administration, and likewise had no effect on the BDNF expression in vHIP, suggesting that BDNF in the ventral hippocampus was involved in the rapid onset of YL-0919. We supposed that increased BDNF synthesis by activating glial sigma-1R might be significant to antidepressant impact of YL-0919. However, there are limitations to the current work that need be considered. As cerebral astrocytes have extensive spatial heterogeneity, explore the genetic and functional changes in different astrocytic subpopulations and brain regions can be investigated. In addition, we will focus on exploring the interplays between astrocytes and other types of cells to explore the development of depression and potential targets for antidepressant drugs.

## 5 Conclusion

This study revealed that YL-0919 exerted a rapid antidepressant effect by regulating mitochondrial function and BDNF levels through astrocytic sigma-1R, which providing new evidence for the discovery of the glial mechanism of rapid antidepressants.

## Data Availability

The original contributions presented in the study are included in the article/supplementary material, further inquiries can be directed to the corresponding authors.
